# Peak ependymal cell stretch overlaps with the onset locations of periventricular white matter lesions

**DOI:** 10.1038/s41598-021-00610-1

**Published:** 2021-11-09

**Authors:** Valery L. Visser, Henry Rusinek, Johannes Weickenmeier

**Affiliations:** 1 Department of Mechanical Engineering, Stevens Institute of Technology, Hoboken, NJ 07030 USA; 2 Department of Biomedical Engineering, Eindhoven University of Technology, 5600 MB Eindhoven, The Netherlands; 3 Institute for Regenerative Medicine, University of Zurich, Zurich, 8006 Switzerland; 4 Department of Radiology, New York University Grossman School of Medicine, New York, NY 10016 USA

**Keywords:** White matter disease, Mechanical engineering, Computational biophysics

## Abstract

Deep and periventricular white matter hyperintensities (dWMH/pvWMH) are bright appearing white matter tissue lesions in T2-weighted fluid attenuated inversion recovery magnetic resonance images and are frequent observations in the aging human brain. While early stages of these white matter lesions are only weakly associated with cognitive impairment, their progressive growth is a strong indicator for long-term functional decline. DWMHs are typically associated with vascular degeneration in diffuse white matter locations; for pvWMHs, however, no unifying theory exists to explain their consistent onset around the horns of the lateral ventricles. We use patient imaging data to create anatomically accurate finite element models of the lateral ventricles, white and gray matter, and cerebrospinal fluid, as well as to reconstruct their WMH volumes. We simulated the mechanical loading of the ependymal cells forming the primary brain-fluid interface, the ventricular wall, and its surrounding tissues at peak ventricular pressure during the hemodynamic cycle. We observe that both the maximum principal tissue strain and the largest ependymal cell stretch consistently localize in the anterior and posterior horns. Our simulations show that ependymal cells experience a loading state that causes the ventricular wall to be *stretched thin*. Moreover, we show that maximum wall loading coincides with the pvWMH locations observed in our patient scans. These results warrant further analysis of white matter pathology in the periventricular zone that includes a mechanics-driven deterioration model for the ventricular wall.

## Introduction

White matter hyperintensities (WMH) are bright appearing white matter tissue lesions in T2-weighted fluid attenuated inversion recovery (FLAIR) magnetic resonance imaging (MRI)^1–3^ and are a frequent observation in the aging human brain^4,5^. WMHs have been linked to vascular degeneration during aging^3,6^ and multiple sclerosis^7,8^. It is typically described that hypertension^9,10^, smoking^11,12^, diabetes^13,14^, and heart disease^10,15^ are common risk factors that exacerbate the vascular involvement leading to white matter changes. WMHs are classified as deep white matter hyperintensities (dWMH) and periventricular white matter hyperintensities (pvWMH) on the basis of anatomical localization^16^. It is well established that cerebral ischemia and small vessel disease are the primary pathophysiological observations in WMHs^3,6,17,18^. These pathologies are insufficient, however, to rationalize the consistent onset of pvWMHs in the horns of lateral ventricles (LV) as opposed to other locations of the ventricular wall. We pose, that the onset of pvWMHs is subject to additional mechanical damage mechanisms. We expect that a lifetime of cyclic mechanical loading of the ependymal cells lining the ventricular wall due to a combination of hemodynamic forces and cerebrospinal fluid (CSF) flow leads to cell damage, structural degeneration of the LV wall, and its progressive functional failure. Here, as a first step, we use a finite element modeling approach to show that ependymal cells (EC) experience peak mechanical loading in the ventricular horns that co-localizes with WMH masks obtained from clinical patient data. The finite element modeling approach is particularly useful because it allows us to create anatomically accurate brain models of various ventricle shapes to demonstrate that EC stretch patterns are consistent across subjects.

Clinically, WMHs are a common observation in medical images of the elderly and their severity typically increases with age^19–21^. By the age of 44, there is a 50.9% likelihood of incidental WMH findings in healthy, cognitively normal subjects^22^ and by age 60, nearly every brain exhibits signs of white matter lesions^23,24^. While early stages of these white matter lesions are only weakly associated with cognitive impairment^25^, the accumulation and expansion of white matter lesions is considered a reliable indicator for long-term functional decline^26,27^. In particular, the volume of pvWMHs is associated with accelerated functional decline^28^.

Pathophysiologically, white matter lesion progression is poorly understood, although there is increasing evidence for location-specific differences. PvWMHs are much more likely to be observed in the aging brain than dWMHs. DWMHs are linked to ischaemic damage, hypoxia, and hypoperfusion^29,30^. Although pvWMHs are associated with ischaemia of perforating arterioles as well, they are further characterized by ependymal cell thinning, leakage CSF into the tissue behind the ventricular wall, and, as inflammatory processes expand, manifests as demyelination, axonal loss, reduced glial density, tissue atrophy^31–34^, and the formation of astroglial scarring^35–37^. Fazekas et al. reported that pvWMHs consistently first appear in the ventricular horns and expand towards deep white matter tissue over time^1^. This observation is reflected in the evaluation criteria proposed by Fazekas et al. yielding a score from 1 to 3. The Fazekas score (FS) differentiates between *caps* or *pencil-thin linings* in the horns associated with the earliest manifestation of pvWMHs (FS=1), *smooth halos* linked to progressively growing pvWMH volumes (FS=2), and ultimately *irregular periventricular signal* that extends into deep white matter (FS=3)^1^. Incidental white matter findings in young healthy adults further support the notion that pvWMHs first emerge in ventricular horns^22^. To date, however, no theory has been proposed to explain the underlying mechanism. Moreover, temporal changes of white matter lesions are poorly understood^38^. White matter lesion volume has been shown to grow on average by 14.6% per year in dWMH and by 9.9% per year in pvWMH^39^. There remain critical knowledge gaps, however, in explaining WMH volume growth over time and the progressive expansion of WMHs along the ventricular wall, on the one hand, and the diffusion of CSF and white matter inflammation radiating out from the ventricular horns into deep white matter on the other.

**Figure 1:**
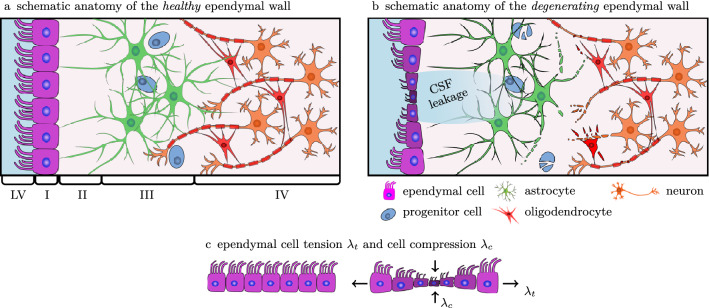
The ventricular epithelium is a functional barrier between the brain and CSF. It consists of four distinct layers: a monolayer of cuboidal multiciliated ependymal cells (Layer I), a prominent hypocellular gap very rich in processes from ependymal cells and astrocytes (Layer II), a ribbon of cells composed of astrocytes (Layer III), and a transitional zone into the brain parenchyma (Layer IV)^40^. (**a**) In a healthy state, the ependymal wall regulates the exchange of fluid and nutrients between the lateral ventricle and brain tissue. (**b**) With age, the thin layer of ependymal cells degrades, leading to the unregulated influx of CSF into the hypocellular layer first and deeper white matter tissue next. Once breached, white matter lesions emerge and propagate. (**c**) Given the morphology of the ventricular wall, ependymal cells are *stretched thin* due to pulsatile fluid flow in the lateral ventricles^32^.

Anatomically, the LVs are lined by the ependymal wall that is composed of distinct layers, see Fig. 1, with varying thicknesses and densities. Going from ventricle towards brain parenchyma, one observes a monolayer of cuboidal multiciliated ependymal cells (Layer I), a prominent hypocellular gap rich in processes from ependymal cells and astrocytes (Layer II), a ribbon of cells composed of astrocytes (Layer III), and a transitional zone into the brain parenchyma (Layer IV)^40^. The multiciliated ependymal cells in Layer I are tightly joined by connexins and cadherins, or gap junction proteins, which form tight inter-cellular connections^33^. Layer II is present in the entire ependymal wall in the LVs, but varies in thickness from region to region. The presence of aquaporin 4 in the basolateral plasma membranes of ependymal cells results in a directed water flow^41^. Aquaporin 4 junctions facilitate the reabsorption of interstitial fluid into the parenchymal vasculature^42^ and drive the unidirectional fluid drainage into ventricular spaces^41^. If the ependyma is disrupted (Fig. 1b), reactive astrocytes will line the ventricular wall in an attempt to reestablish homeostasis^33,35^. The reactive replacement of ependymal wall with gliotic tissue is associated with the lack of polarized aquaporin 4 junctions. This leads to an undirected transport of CSF through the ependyma^34,35^. Over time, the dysregulated CSF-brain parenchyma barriers leads to excessive influx of CSF into the hypocellular layer and the accumulation of fluid and dysregulated homeostasis^43^. This mechanism provides a rationale for the deterioration of periventricular ependymal cell damage into deep reaching progressive white matter lesions.

The primary objective of the present work is to show that peak ependymal cell loading co-localizes with pvWMHs along the ventricular wall. We juxtapose subject-specific finite element simulations of ventricular wall loading and FLAIR image-derived WMH masks to test our proposed white matter lesion model. Previous computational models of the (peri)ventricular space primarily focused on assessing hydrocephalus^44–48^ or white matter damage during traumatic brain injury^49^. These models elucidate organ-level mechanical tissue behavior, but do not reveal the cellular loading state caused by hemodynamic loading and CSF flow in the LVs. Here, we selected our subjects based on a broad representation of ventricular shapes in order to demonstrate that the mechanical loading of ependymal cells along the ventricular wall consistently peaks in the horns. The progressive deterioration of initially small caps into deep-penetrating pvWMHs is likely caused by subsequent damage mechanisms that involve vascular and mechanical contributions. We therefore suggest that the mechanical loading of ependymal cells is one of the most important risk factors for the emergence of pvWMHs.

## Methods

### Subject selection and WMH segmentation

We obtained magnetic resonance images (MRI) from cognitively normal subjects in the imaging database of the New York University Alzheimer’s Disease Research Center (NYU ADRC). The study was approved by the NYU Langone Institutional Review Board. Each subject provided written informed consent for a protocol investigating risk factors of cognitive decline and Alzheimer’s disease. All procedures performed in studies involving human participants were in compliance with the ethical standards of the Health Insurance Portability and Accountability Act and with the 1964 Helsinki Declaration and its later amendments. Subjects’ clinical evaluation included an interview according to the Brief Cognitive Rating Scale and rating on Global Deterioration Scale (GDS)^50^. Subjects with brain pathology such as tumor, neocortical infarction, multiple sclerosis, and diabetes were excluded. Also excluded were those using psychoactive medications and subjects scoring < 16 on the 17-item Hamilton Depression Scale^51^. This selection yielded a subset of N = 352 cognitively healthy elderly: 209 women, 68.1 ± 8.0 year old (mean ± standard deviation) and 143 men, age 71.8 ± 7.3. Based on clinical assessment, all subjects in the subset were diagnosed as cognitively healthy, i.e. with GDS = 1, i.e., no subjective memory complaints, or GDS = 2, i.e., with subjective memory complaints, but not fulfilling the criteria for mild cognitive impairment or dementia. All selected subjects had scored at least 27 points on the Mini Mental State Examination^52^.

**Figure 2:**
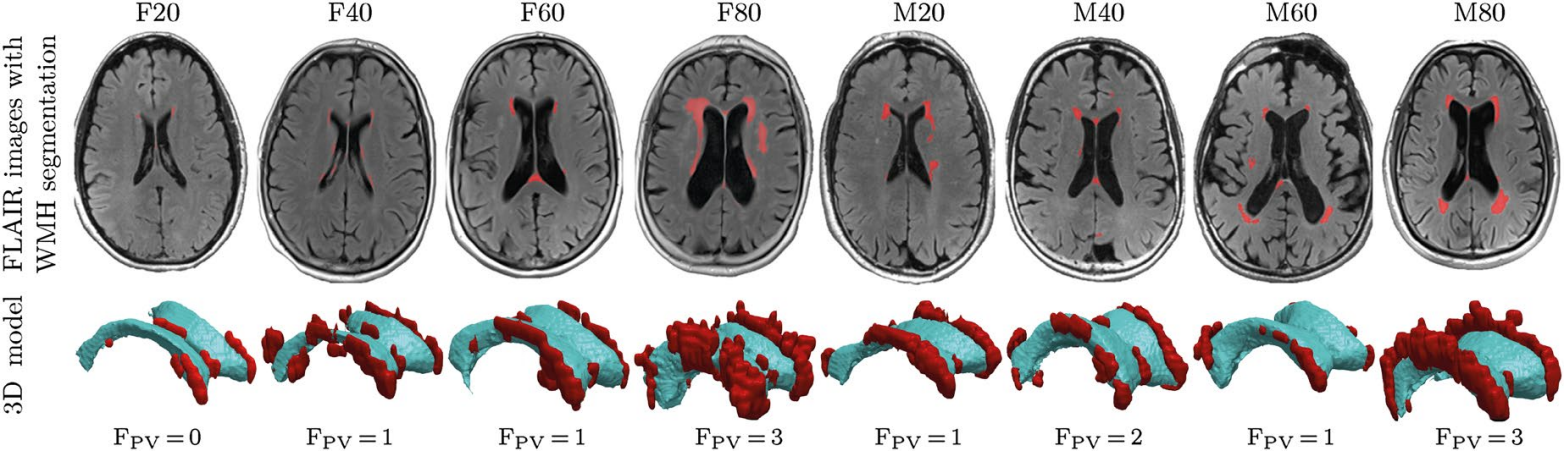
Representative axial slices and volumetric reconstructions of lateral ventricles and white matter hyperintensities from eight subjects. We sorted 464 subjects from the NYU ADRC’s imaging database based on their total intracranial CSF volume and picked a male and female subject from the 20th, 40th, 60th, and 80th percentile. The top row shows the FLAIR MRI sequence of all eight patients with a white matter hyperintensity mask (red); the bottom row shows volumetric reconstructions of their ventricles (blue) and white matter hyperintensities (red). We also provide the Fazekas score of the periventricular WMHs.

Each subject underwent structural MRI on a 3T Siemens Magnetome Prisma (Siemens Healthineers USA). The exam included a high-resolution T1-weighted MPRAGE sequence (TR = 2100 ms, TE = 5 ms, TI = 900 ms, FA = 9$^{\circ }$, 256 × 256 × 176 matrix, 1 × 1 × 1 mm voxels, GRAPPA2 acceleration) and a FLAIR sequence used to assess WM lesions (TR = 9000 ms, TE = 75 ms, TI = 2500 ms, FA = 120$^{\circ }$, 320 × 196 × 40, 0.7 × 0.7 × 4 mm voxels, GRAPPA2 acceleration). MPRAGE images were segmented into gray matter, white matter, and cerebrospinal fluid using Statistical Parametric Mapping Version 12 implemented in Matlab^53^. We sorted subjects by gender and based on their total intracranial CSF volume^54^. To capture a broad range of ventricular geometries, we selected male and female subjects from the 20th, 40th, 60th, and 80th percentile of total intracranial CSF volume, as shown in Fig. 2, which are labeled as F20/F40/F60/F80 (females) and M20/M40/M60/M80 (males), respectively. Our eight subjects are on average 73.4$\,\pm$ 5.9 years of age; their Fazekas score was assessed separately by two neurologists from the New York Langone Medical Center; and CSF and LV volume were derived from Freesurfer segmentations of the MPRAGE images. From a mechanics perspective, eight subjects is a reasonable sample size to demonstrate the repeatable ependymal cell loading state along the ventricular wall across a wide range of ventricular shapes. For all eight subjects, WMHs were segmented on FLAIR images with FireVoxel (build 301, www.firevoxel.org). The fully automatic algorithm starts with uniformity correction^55^, followed by the estimation of the signal intensity within an image-dependent whole-brain mask $\Omega$. The WHMs were then segmented by thresholding from $\Omega$ all voxels *v*, such that $\bar{\mathcal {M}}\,=\,\{v\,|\, v \in \Omega \wedge s(v)>\mu \,+\,k\,\sigma \}$, where $\mu$ is the mean value and $\sigma$ the standard deviation of intensity distribution in $\Omega$, and *k* was set at 2.5. The aim of the final step is to delete from $\bar{\mathcal {M}}$ the septum and choroid plexus. These structures are identified as connected components of $\bar{\mathcal {M}}$ having > 50% surface boundary adjacent to CSF. The resulting WMH masks $\mathcal {M}$ were quality-controlled by experienced neuroscientists from the AD Center.

### Finite element model generation

**Figure 3:**
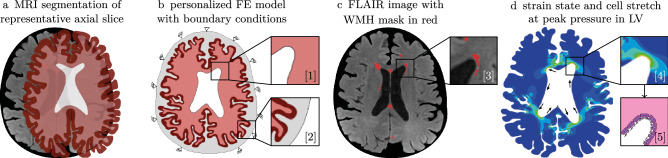
We generated finite element models for each subject individually by segmenting axial MRI through the lateral ventricle. (**a**) We identified the boundaries of gray matter, white matter, lateral ventricles, and cerebral spinal fluid surrounding the cortex. (**b**) The model was kinematically constrained along the outer boundary, and we applied a pressure normal to the ventricular wall (b1) and normal to the outer gray matter surface (b2). (**c**) Accompanying FLAIR images superposed with a mask of white matter hyperintensities show the localization of early leukoaraiosis in the ventricular horns (c3). We simulated peak loading during the hemodynamic pressure cycle in order to obtain the strain field (d4) and ependymal cell stretch along the ventricular wall (d5).

We generated personalized finite element models of each subject based on a semi-automatic segmentation approach. We segmented each structural MR image using Freesurfer and imported both, the structural scan and the Freesurfer segmentation, into the 3D image processing and model generation software Simpleware$^{TM}$ (Synopsys, Inc., Mountain View, CA)^56–58^. We identified the axial slice with the largest ventricular area showing the anterior and posterior horns and manually corrected the imported Freesurfer segmentation of the lateral ventricle, white and gray matter, and surrounding CSF based on the co-registered structural scan. Figure 3 shows the model generation process. Using the FE Module of Simpleware, we converted our segmentations into finite element meshes and imported our models into Abaqus (Dassault Systémes, Providence, RI). We constrained our model against out-of-plane deformations (plane strain simulation) and applied pressure boundary conditions to two surfaces: a normal pressure to the ventricular wall (green arrows in Fig. 3b1) and a normal pressure to the gray matter-CSF interface (blue arrows in Fig. 3b2). These loading conditions represent the cyclic brain deformations caused by a combination of hemodynamic forces and CSF flow^59^. The lateral ventricles, in particular, undergo a piston-like motion causing cyclic volumetric expansion during systole^60^. To that end, we prescribed an LV pressure of 20 Pa, or 0.15 mmHg, and a subarachnoid space (SAS) pressure of 1 Pa, or 0.0075 mmHg, in line with observations that SAS pressure is only 10-20% of the ventricular pressure^61–64^. We mimic the skull, which is orders of magnitudes stiffer than cerebral tissues, by prescribing zero-displacement boundary conditions, shown in gray in Fig. 3, on the outer periphery of our model. We do not model fluid flow around the brain as well as fluid flow and CSF production in the ventricles, but assume fluid cavities to expand and contract with each heartbeat. Therefore, we approximate CSF as an ultrasoft, compressible material with a Young’s modulus of 0.1 kPa and a Poisson’s ratio of 0.3. Brain tissues were modeled as an Ogden-type hyperelastic material model^65,66^. Following basic continuum theory of finite deformations, we introduce the deformation gradient $\mathbf {F}$ as the gradient of the nonlinear deformation field $\phi$ with respect to the material coordinates $\mathbf {X}$ in the reference configuration. Assuming nearly incompressible behavior of brain tissue, we decompose the deformation gradient $\mathbf {F}$ into a volumetric contribution characterized through the Jacobian *J* and an isochoric contribution $\bar{\mathbf {F}}$,

1 $$\begin{aligned} \mathbf {F} = \nabla _\mathbf {X}\mathbf {\phi } = J^{1/3} \bar{\mathbf {F}},\,\,\,\text {with}\,\,\, J = \det (\mathbf {F}) \,\,\,\text {and}\,\,\, \bar{\mathbf {F}} = J^{-1/3}\mathbf {F}. \end{aligned}$$

As a characteristic deformation measure, we introduce the right Cauchy-Green deformation tensor $\mathbf {C}$ which obeys a similar decomposition into a volumetric contribution in terms of the Jacobian *J* and an isochoric contribution ${\bar{\mathbf{C}}}$,

2 $$\begin{aligned} \mathbf {C}=\mathbf {F}^T\cdot \mathbf {F}=J^{2/3}\bar{\mathbf {C}},\,\,\text {with}\,\,\, J^{2/3}=\text {det}^{2/3}(\mathbf {F})\,\,\,\text {and}\,\,\,\bar{\mathbf {C}}=\bar{\mathbf {F}}^T\bar{\mathbf {F}}. \end{aligned}$$

We can then introduce the isochoric first and second invariants, $\bar{I}_1$ and $\bar{I}_2$, either in terms of the isochoric right Cauchy-Green deformation tensor ${\bar{\mathbf{C}}}$, or in terms of the isochoric principal stretches $\bar{\lambda }_1$, $\bar{\lambda }_2$, and $\bar{\lambda }_3$, recalling that $\bar{I}_3 = J^2 = 1$,

3 $$\begin{aligned} \begin{aligned} \bar{I}_1&=\text {tr}(\bar{\mathbf {C}})=\bar{\lambda }_1^2+\bar{\lambda }_2^2+\bar{\lambda }_3^2,\\ \bar{I}_2&=\frac{1}{2}[\text {tr}^2(\bar{\mathbf {C}})-\text {tr}(\bar{\mathbf {C}}^2)]=\bar{\lambda }_1^2\bar{\lambda }_2^2+\bar{\lambda }_2^2\bar{\lambda }_3^2+\bar{\lambda }_3^2\bar{\lambda }_1^2. \end{aligned} \end{aligned}$$

It has been shown that the mechanical response of brain tissue is best captured by a one-term Ogden model given by the strain energy density function, $\Psi$,^65^

4 $$\begin{aligned} \Psi =\frac{\mu }{2}[\bar{\lambda }_1^2+\bar{\lambda }_2^2+\bar{\lambda }_3^2-3]+\frac{\kappa }{4}[J^2-1-2\,\log (J)], \end{aligned}$$

with shear modulus $\mu$ governing isochoric, distortional deformations and bulk modulus $\kappa$ governing dilatational deformation. We assume our material to be nearly incompressible with a Poisson’s ratio of 0.45 and a white-gray matter stiffness ratio of 2^65^. Specifically, we chose experimentally-informed constants $\mu =0.34$ kPa and $\kappa =3.3$ kPa for gray matter and $\mu =0.68$ kPa and $\kappa =6.6$ kPa for white matter^61,65,67–69^. We implemented the Ogden model in a user material subroutine (UMAT) following the example of Connolly et al.^66^ and compute ventricular wall stretches as outlined further below.

### Mechanomarkers for ventricular wall loading

Our proposed damage mechanism rests on the premise that the cuboidal multiciliated ependymal cells lining the ventricular wall experience a mechanical loading state that compromises their integrity. Specifically, we submit that the tight connections between ependymal cells formed by cadherin junctions are particularly vulnerable to mechanical deformations and that their increased loading ultimately leads to failure^32,33^. The subsequent leakage of CSF into the surrounding white matter appears as hyperintensities in FLAIR images. To that end, we determine cellular deformations and differentiate between ependymal cell tension in the direction tangential to the ventricular wall and ependymal cell compression in the direction normal to the ventricular wall, see Fig. 3d5. We determine the normal and tangential wall directions from a Laplacian diffusion simulation for each of our eight FE models. We prescribe a temperature boundary condition of 1.0 $^{\circ }$C on the nodes outlining the ventricular wall, 0.2 $^{\circ }$C on the interface between gray and white matter, and of 0.0 $^{\circ }$C for all nodes on the interface between gray matter and CSF. The resulting flux field allows us to identify the directions of the steepest temperature gradient $\mathbf {n}_0$, normal to the undeformed ventricular wall, and isothermes $\mathbf {t}_0$, tangential to the undeformed ventricular wall. We import the two vector fields into our simulations and project the right Cauchy-Green deformation tensor $\mathbf {C}$ onto these directions to obtain cell tension and cell compression. Specifically, we calculate the right Cauchy-Green strain tensor and project strains onto the normal and tangential directions, respectively,

5 $$\begin{aligned} \lambda _{\text {t}}=\sqrt{\mathbf {t}_0\cdot \mathbf {C}\,\mathbf {t}_0}\quad \text {and}\quad \lambda _{\text {c}}=\sqrt{\mathbf {n}_0\cdot \mathbf {C}\,\mathbf {n}_0}. \end{aligned}$$

During post-processing of each simulation, we identify the nodes lining the ventricular wall, starting at the midpoint between the two posterior horns of the lateral ventricle and move counter-clockwise along the ventricular wall, see Fig. 3d5. We then determine ependymal cell tension and ependymal cell compression in each node to arrive at the representations shown in Fig. 5. Furthermore, we compute the maximum principal strains (MPS) from the Green-Lagrange strain field given by $\mathbf {E}=1/2\,(\mathbf {C}-\mathbf {I})$, with identity tensor $\mathbf {I}$. As shown in Fig. 5, MPS is a representative measure for tissue loading in the vicinity of the ventricular wall and takes on a similar outline as the WMH mask.

To quantify geometric differences between our subjects’ lateral ventricles, we introduce two measures that describe horn shape and the degree of wall loading. For each of the four horns, we fit a circle through three points: the location of maximum cell tension, and the two locations left and right of this point where cell tension has dropped to 10% of the maximum value. We measure the radius *r* of the sphere as a critical measure for horn geometry. We also determine the ventricular wall sections that are exposed to cell stretches above 10% of the maximum value. Specifically, we measure the length of the ventricular wall exposed to this critical cell stretch for each horn and divide this measure by the total wall length.

### Statistical analysis

Statistical analysis was performed using Matlab. Results are reported as mean ± standard deviation. We fitted CSF volume, WMH volume, peak EC stretch, and wall fraction under elevated EC stretch against horn radius using least square optimization and report R$^2$ values, Pearson’s correlation coefficient $\rho$, and *p*-value. We used a two-sample T-test to determine weather EC stretch spatially correlates with pvWMH location. To obtain an independent data set, we sample values of the parameters at 30 equidistant points along the ventricular wall. We differentiate between parameter values at points where pvWMHs are present (n = 41) and compare them to the parameter values where there are no pvWMHs (n = 199). We report the test statistic, number of degrees of freedom, and *p*-value.

## Results

### Morphological changes of the lateral ventricles

Table 1 lists age, CSF, LV, WMH volumes, and Fazekas score for each subject. CSF, LV, and WMH volumes were determined via FireVoxel^70^. CSF volumes range from 421 cm$^3$ (20th percentile) to 581 cm$^3$ (80th percentile). Lateral ventricle volumes (vLV) range from 14 cm$^3$ (20th percentile) to 74 cm$^3$ (80th percentile) with an average vLV of 45.7±16.2 cm$^3$. WMH volumes range from 1.1 cm$^3$ to 20.7 cm$^3$ with an average volume of 8.55±6.6 cm$^3$. In spite of a small sample size we observe significant correlations between lateral ventricle volume and Fazekas score (Pearson’s correlation coefficient r(8) = 0.77, *p* = 0.026) as well as WMH volume and Fazekas score (r(8) = 0.95, *p* < 0.001). In males, WMH volume is 1.2 times larger than in females with a mean WMH volume of 9.4 cm$^3$ for male subjects and 7.8 cm$^3$ for female subjects. Figure 2 shows WMH caps around the anterior horns for F/M20, smooth WMH halos for F/M40-60, and increasingly diffuse and deep-reaching WMHs for F/M80.

**Table 1:** Summary of subject data including age, CSF volume (vCSF), lateral ventricle volume (vLV), WMH volume (vWMH), and Fazekas score (FS).

Subject	Age (year)	vCSF (cm$^3$)	vLV (cm$^3$)	vWMH (cm$^3$)	FS
F20	65.1	421.0	30.2	1.1	0
F40	68.5	473.0	34.2	5.2	1
F60	75.3	519.0	40.1	4.0	1
F80	77.6	579.0	74.1	20.7	3
M20	68.5	451.0	33.4	6.2	1
M40	81.1	493.0	35.3	7.8	2
M60	79.9	519.0	56.9	7.0	1
M80	71.4	581.0	61.4	16.4	3

We selected eight representative cognitively normal subjects from the 20th, 40th, 60th, and 80th percentile of CSF volume.

**Figure 4:**
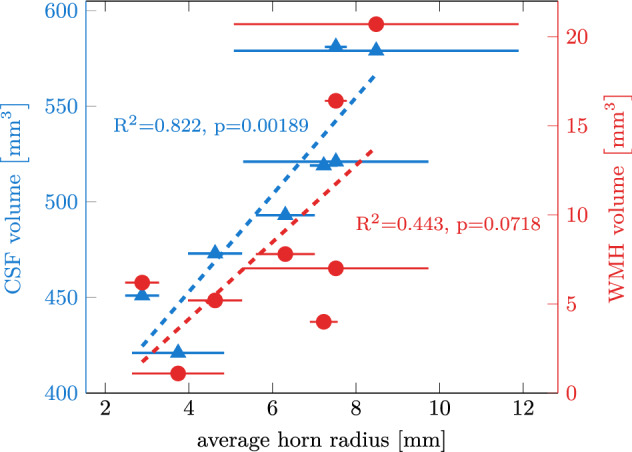
Horn radius versus CSF volume and WMH volume. We show the average of all four horn radii for each subject (marker with standard deviation as a horizontal line). We observe an overall increase of horn radius with increasing CSF and WMH volumes.

When we fitted a circle into each of the horns in our eight models, we observe that horn radius consistently increases with vCSF and vWMH. Figure 4 shows the relationships between averaged horn radius per subject (markers show mean horn radius with horizontal lines indicating the standard deviation) and CSF (blue data) and WMH volumes (red data). Based on a linear fit through both data sets, we observe an R$^2$ value of 0.822 with *p* = 0.00189 for vCSF and an R$^2$ value of 0.443 with *p* = 0.0718 for vWMH. An increasing horn radius is a characteristic morphological manifestation of aging brains which undergo significant cerebral atrophy and ventricular enlargement^10,31,71–73^.

### Ependymal cell stretch along the ventricular wall

**Figure 5:**
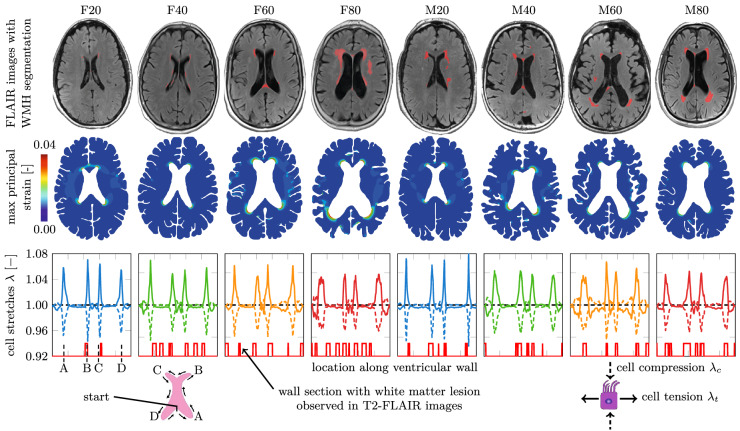
Eight finite element models were created from FLAIR MRI with WMHs shown in red (top row). We simulated quasistatic ventricular expansion during peak loading to determine ventricular wall strain and ependymal cell stretch. The distribution of maximum principal strain (middle row) is consistent across all individuals, with peak strain localizing around the anterior and posterior horns of the lateral ventricles. Based on the deformation fields, we determine ependymal cell stretch along the ventricular wall. We calculate ependymal cell tension, which is tangential to the ependymal wall, and ependymal cell compression, which is perpendicular to the ependymal wall. We observe four distinct locations with maximum stretches along the wall. These coincide with the horns, as indicated by points A, B, C, and D. We report both stretch components for every point along the ependymal wall as shown in the ventricular representation in the legend.

Figure 5 shows the WMH mask in FLAIR images of our eight subjects, the maximum principal strain, and the computed ependymal cell stretches (EC stretch), associated with cell tension (elongation) and cell compression (thinning), see Fig. 1c. The maximum principal strain field is highest at the edge of the ventricles and diffuses towards deeper white matter. We observe a significant increase in maximum EC stretch in ventricular horns, with maximum cell tension of up to 1.07 in female and 1.08 in male subjects; maximum thinning occurs in the same locations where we observe cell compression of up to 6% in female and 7% in male subjects. All eight subjects show a similar pattern of four sharp focal points with significantly increased cell stretches in both tension and compression. The locations of the four focal points coincide with the anterior and posterior horns of the lateral ventricles. A two-sample T-test demonstrated that EC stretch is significantly higher in the presence of pvWHMs in comparison to where there are no pvWMHs with t(49) = 3.58 and *p* = 0.00039.

### Impact of ventricular geometry on ependymal cell loading

**Figure 6:**
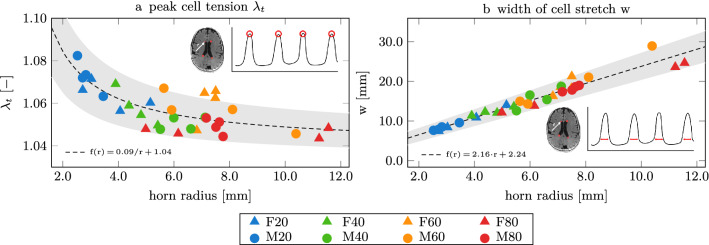
Ventricular geometry is an indicator for peak cell stretch (a) and the wall fraction exposed to elevated cell stretch (b). We measure the radius of a sphere fitted to the anterior and posterior horns as a representative marker for ventricular geometry. We observe that peak cell stretch decreases for increasing horn radii and that an increasing wall section experiences elevated cell stretch as horn radius increases. Our observations suggest that younger brains with sharper ventricular horns, i.e., smaller horn radii, experience higher ependymal cell loading while aged brains (with larger horn radii) experience lower cell loads but elevated stretches on larger wall sections, i.e., increased width of cell stretch.

Despite the consistency of cell stretch patterns along the ventricular wall in our subjects shown in Fig. 5, we observe distinct changes in the mechanical loading state with increasing horn radius. As such, subjects with larger ventricular volume consistently have larger horn radii in comparison to subjects with smaller ventricles. Mechanically, curvature of the ventricular wall, $\kappa$, is inversely proportional to horn radius *r* with $\kappa \,=\,1/r$ and directly correlates with cell tension and cell compression. This relates to the observation that as ventricular horns enlarge, cellular stretch magnitude decreases, but the region of elevated cell stretch increases in comparison to sharp peaks in younger brains. Figure 6 shows how peak cell stretch (Fig. 6a) and wall fraction with elevated cell tension (Fig. 6b) vary as a function of horn radius. As ventricular volume increases, horn radii increase as well. We observe that peak EC stretch drops from 1.085 to 1.04 for increasing horn radius. More strikingly, after fitting a reciprocal function for peak cell stretch as a function of horn radius, we obtained the functional relationship $f_{\lambda _t}(r)=0.09/r+1.04$ (R$^2$ = 0.59) and a Pearson’s correlation coefficient of $\rho$ = – 0.68 (*p* < 0.001), despite our small sample size. The ventricular wall fraction affected by elevated EC stretch increases with horn radius by up to a factor 3. After fitting a linear function for wall fraction as a function of horn radius, we obtained the functional relationship $f_w(r)=2.16\cdot r+2.24$ (R$^2$ = 0.93) and a Pearson’s correlation coefficient of $\rho$ = 0.97 (*p* < 0.001).

### Sensitivity analysis of model parameters

**Figure 7:**
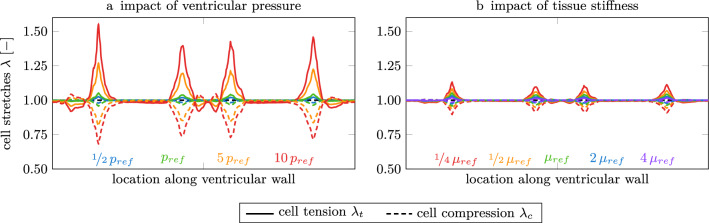
Sensitivity analysis of ventricular pressure level (a) and mechanical brain tissue stiffness (b) uncovers the impact on ependymal wall stretches. A ten-fold increase in referential ventricular pressure leads to cell stretch of up to 1.56 and cell compression as low as 0.71. In comparison, a four-fold increase in tissue stiffness reduces peak cell stretches by a factor 0.97 and a four-times lower shear modulus leads to a 1.07-fold increase in peak cell stretch. (All values are relative to tissue stiffness reported in the “Methods” section). Ventricular pressure is highly heterogeneous across subjects^76^. As discussed in the next section, ventricular pressure appears to be the critical parameter in the etiology of ependymal cell fatigue.

We analyzed our model’s sensitivity to ventricular wall pressure and white matter tissue stiffness, and show the results for subject F40 in Fig. 7. We applied intracranial pressures of 0.5, 5, and 10 times the referential pressure of 20 Pa and observe a significant increase in the average peak cell stretch value of up to 1.56. Specifically, when applying 5-times the referential pressure, maximum cell tension increases by a factor of 1.21 and maximum cell compression increases by a factor of 1.18; when applying 10-times the referential pressure, maximum cell tension increases by a factor of 1.48 and maximum cell compression increases by a factor of 1.41. Strikingly, we observe a minimal effect on the lateral sides of the ventricular cavity and no shift in the location of peak stretches. The wall fraction exposed to increased cell tension increases with increasing pressure while the location of peak load remains unchanged. The range of ventricular pressures was based on literature reporting significant pressure variations for healthy subjects and subjects with hypertension and hydrocephalus^74–76^.

We simulated variable tissue stiffness and compared cell stretches for white matter stiffness ranging from 25% to 400% of the original, experimentally observed stiffness to represent the range of values reported in literature^65,68,77^. In comparison to the variation in pressure, we observe a significantly lower effect, with a maximum 1.07-fold increase in peak cell loading, see Fig. 7b. It is important to note, however, that tissue softening leads to increased cell loads. This provides evidence that tissue degeneration is a mechanically driven mechanism that leads to accelerated tissue aging and pvWMH volume growth.

## Discussion

### Patient-specific simulation of ventricular wall loading

We determine ependymal cell tension (tangential to wall) and ependymal cell compression (normal to the wall) by projecting the right Cauchy-Green deformation tensor onto the principal directions of the ependymal cells lining the ventricular wall. Figure 5 (bottom row) shows that despite pronounced variations in lateral ventricle geometry across our subjects, peak cell tension and cell compression consistently localize in the anterior (B, C) and posterior (A, D) horns. Comparison with the location of pvWMHs along the ventricular wall, reveals that tissue damage co-localizes in regions of peak cell stretches. The loading condition in these regions leads to ependymal cells being *stretched thin* and this is expected to impose a particularly high load on intercellular cadherin junctions^33^. The exterior sides of the ventricular wall (between points A and B, and between points C and D) experience negligible cell stretches. The anterior and posterior attachment points of the septum pellucidum (the wall section between points D and A, and between points B and C) exhibit slightly elevated stretches and cause cell compaction, i.e. the opposite loading state than in the horns. Therefore, we pose that the clinical observation of pvWMHs first appearing as localized caps in the anterior and posterior horn is in part driven by the particular mechanical loading state of ependymal cells^35^. Previous studies have shown that once initiated, pvWMHs deteriorate into bright-appearing linings and ultimately deeper reaching WMHs as LV wall failure worsens and CSF diffuses into deeper white matter regions^32,33,43^. Future work could provide a constitutive model for pvWMH volume growth and the progressive deterioration of periventricular tissue due to the diffusion of CSF into deep WM structures.

### Ventricular deterioration changes ependymal cell loading

Mechanically, horn radius provides a marker for the severity of cellular loading. Biologically, horn radius increases with age due to ventricular enlargement caused by tissue degeneration. These observations are critical in understanding the impact of mechanical loading on the progressive deterioration of the ependymal wall^10,31^. Younger, healthy brains are generally associated with smaller ventricles, sharper horns, and higher cell stretches. As ventricular volume increases with age, horn radius increases as well and causes a growing wall section to be exposed to elevated EC stretches. It is expected that with age, ependymal cells and their tight cadherin junctions experience mechanical fatigue due to lifelong hemodynamic loading. Accompanying cellular deterioration leads to CSF leakage into periventricular white matter and facilitates secondary tissue damage mechanisms in layers II-IV, see Fig. 1. We pose that horn radius may be a reliable biomarker for the clinical assessment of a subject’s risk of developing pvWMHs or leukoaraiosis^71^.

### Risk factors for the onset of periventricular white matter lesions

Hydrocephalus and other neurological diseases that lead to increased intracranial and blood pressure pose a significant risk to the mechanical integrity of the ventricular wall^78–80^. It has been shown experimentally that a rapid increase in ventricular pressure leads to ependymal wall failure and the diffusion of CSF into white matter tissue^43^. Strikingly, this was observed to occur first in the ventricular horns, which agrees with our hypothesis that ependymal cells experience peak mechanical load in these locations which leads to functional failure. Therefore, our unifying physics-driven damage model explains pvWMHs locations and is supported by histopathological studies on ventricle surface gliosis^81^ and the observation of decreased white matter integrity^82^. Moreover, our model and damage hypothesis provide a rational for the different stages of pvWMHs as they evolve in parallel with the extended strain field observed in our simulations. Our sensitivity analysis revealed, that subjects with increased intracranial or hemodynamic pressure are at an elevated risk to damage their ventricular wall and are more likely to develop leukoaraiosis^19^. Clearly, aging and small vessel disease are important risk factors as well. Peak wall loading in the form of ependymal cell thinning occurs in the horns and extends radially into deeper white matter. Lateral sides of the ventricles are exposed to negligible loads and are subsequently more protected^81^. Once the ventricular wall is disrupted, ependymal wall layers II-IV experience CSF influx and tissue damage due to inflammation and astrogliosis^32,34^. It was shown that astrocytes cover the denuded ventricular walls of hydrocephalic hyh mutant mice to form a new cell layer with a cell organization that resembles the ependyma^38^. The fluid accumulation in the hypocellular layer triggers subsequent damage mechanisms such as astroglial scarring and the depletion of progenitor cells, which are required for tissue regeneration^37,38^. PvWMHs first appear as caps in the ventricular horns and are independent of a subject’s ventricular shape at the time of onset. Although vascular damage is a driving force in WMHs, the consistent localization in horns suggests the involvement of additional factors. Based on our mechanics-driven hypothesis, sharp horns, i.e., small horn radii, cause high ependymal cell loads and therefore represent a critical additional risk factor for wall failure.

### Mechanomarkers for periventricular white matter hyperintensities

Our numerical modeling approach shows good agreement between EC stretch and pvWMH location for early stages of pvWMH formation, i.e., models M/F20 and M/F40. As pvWMHs increase and penetrate into deeper white matter regions, our current model requires additional mechanisms to propagate tissue deterioration from the ventricular wall into deeper layers of the periventricular zone. Specifically, our model requires a constitutive damage mechanism to quantify the degree of ependymal cell fatigue in order to trigger local tissue softening and the subsequent expansion of the critically loaded wall segments^83^. Distinct structural brain changes, such as cerebral atrophy^84,85^ or white matter pathology^86^, are useful biomarkers for diagnosis of abnormal aging. Our model establishes that ependymal cell loading might be a mechanomarker for pvWMH location. Cell loading magnitude is directly affected by ventricular pressure and LV shape. Therefore, identification of subjects with critical ventricular geometry, i.e. small horn radii, or increased cardiovascular risk would allow for early intervention via anti-hypertensive therapy^87^. Previous computational models of hydrocephalus have observed elevated interstitial pressure and tissue stress concentrations in the posterior and anterior horns as well^44,45,48^. While these models appear similar in the simulation approach, our primary interest in quantifying the cellular stretches of ependymal cells forming the ventricular wall is distinctly different.

### Limitations

Our sample size of eight subjects is too small to derive age- and LV-dependent trends for pvWMH volume progression. However, our subjects were selected with the goal to demonstrate that EC loading consistently peaks in the ventricular horns and, therefore, correlates with pvWMH locations. As a next step, we will investigate longitudinal changes of pvWMH volume based on a significantly larger sample size. Furthermore, lateral ventricles undergo three-dimensional deformation during the hemodynamic cycle^49^; here, however, we use two-dimensional models based on a representative axial slice showing the largest cross-sectional area of the lateral ventricles. Even though it can be expected that 2D and 3D models will provide minor differences in the numerically predicted deformation field, the peak EC stretches will always appear in locations with high curvature, see Fig. 6. Future work should aim at generating volumetric models to assess the full deformation field of the lateral ventricles. Lastly, we assess EC stretch via a quasi-static loading case instead of studying the dynamic effects of CSF flow in the ventricles and pulsatile motion from the hemodynamic cycle. The lack of in vivo data on the ventricular wall motion, however, is a major limitation to validating a dynamic simulation^47^. To further establish the role of mechanics in the onset and progression of pvWMHs, future work should look into the quantification of in vivo wall motion via novel MRI techniques^59^ and a histological analysis of the ventricular wall’s temporal decay.

## Conclusions

Our model is a first step in formulating a unifying theory for a physics-driven mechanism that explains the onset location of pvWMHs in the aging brain. We observe a mechanical loading state of the ventricular wall that causes ependymal cells to be *stretched thin* during each hemodynamic cycle. This particular loading state represents a major risk factor for cellular damage and functional and structural failure of the ventricular wall as it is repeatedly observed in pathology and histological studies on wall damage in aged brains. While vascular damage is a key contributor to pvWMHs, the consistent initial appearance as caps in the ventricular horns, is strongly indicative of a mechanical contribution to ventricular wall failure. We therefore suggest further investigation into the concise etiology and progression of pvWMHs and its prevention strategies.
